# Experimental Evolution on a Wild Mammal Species Results in Modifications of Gut Microbial Communities

**DOI:** 10.3389/fmicb.2016.00634

**Published:** 2016-05-04

**Authors:** Kevin D. Kohl, Edyta T. Sadowska, Agata M. Rudolf, M. Denise Dearing, Paweł Koteja

**Affiliations:** ^1^Department of Biological Sciences, Vanderbilt UniversityNashville, TN, USA; ^2^Institute of Environmental Sciences, Jagiellonian UniversityKraków, Poland; ^3^Department of Biology, University of UtahSalt Lake City, UT, USA

**Keywords:** artificial selection, gut microbiome, herbivory, host-microbe interactions, voles

## Abstract

Comparative studies have shown that diet, life history, and phylogeny interact to determine microbial community structure across mammalian hosts. However, these studies are often confounded by numerous factors. Selection experiments offer unique opportunities to validate conclusions and test hypotheses generated by comparative studies. We used a replicated, 15-generation selection experiment on bank voles (*Myodes glareolus*) that have been selected for high swim-induced aerobic metabolism, predatory behavior toward crickets, and the ability to maintain body mass on a high-fiber, herbivorous diet. We predicted that selection on host performance, mimicking adaptive radiation, would result in distinct microbial signatures. We collected foregut and cecum samples from animals that were all fed the same nutrient-rich diet and had not been subjected to any performance tests. We conducted microbial inventories of gut contents by sequencing the V4 region of the 16S rRNA gene. We found no differences in cecal microbial community structure or diversity between control lines and the aerobic or predatory lines. However, the cecal chambers of voles selected for herbivorous capability harbored distinct microbial communities that exhibited higher diversity than control lines. The foregut communities of herbivorous-selected voles were also distinct from control lines. Overall, this experiment suggests that differences in microbial communities across herbivorous mammals may be evolved, and not solely driven by current diet or other transient factors.

## Introduction

Animals maintain diverse gut microbial communities that provide a number of physiological functions to their hosts (McFall-Ngai et al., 2013) such as modulating host energy balance (Semova et al., 2012), immune function (Round and Mazmanian, 2009), and behavior (Heijtz et al., 2011). Recent research efforts have focused on understanding interactions between hosts and microbes. Comparative studies have demonstrated that diet, gut anatomy, and phylogenetic history interact to sculpt the gut microbial community structure of mammals (Ley et al., 2008; Muegge et al., 2011). Additionally, experimental studies, particularly those with germ-free rodents lacking gut microbes, have revealed that gut microbes impact the behavior (Heijtz et al., 2011), energy storage, and metabolic rates of animals (Bäckhed et al., 2004). However, sterile animals are highly unnatural, and so it is difficult to conclude whether these differences are relevant in an evolutionary sense.

Selection experiments represent a bridge between these two methods of study and have the potential to test hypotheses and validate conclusions generated by prior comparative studies (Garland, 2003). Selection experiments generally focus on whole-organism traits, such as physiological performance or behavior, and breed the top performing individuals to enhance the trait in subsequent generations. Then, further investigations can be conducted to determine the underlying mechanisms that might drive differences in whole-organism traits. For example, selection for voluntary wheel-running behavior in mice is coupled with alterations in limb bone morphology (Wallace et al., 2012), higher circulating levels of stress hormones (Girard and Garland, 2002), enhanced skeletal muscle glucose uptake (Dumke et al., 2001), and altered gene expression in the brain (Kelly et al., 2012). These studies highlight that selection for whole-organism traits may be driven by changes at many levels of biological organization (Garland, 2003). The gut microbiota is heritable (Funkhouser and Bordenstein, 2013; Goodrich et al., 2014), thought to influence host evolution (Sharon et al., 2010; Brucker and Bordenstein, 2012), and is regularly implicated as an underlying mechanism of differential host phenotypes. However, to date no selection experiments have investigated connections between selection on whole-organism traits and concomitant changes in gut microbial community structure. Filling this knowledge gap is critical, especially given new interest in the “hologenome concept,” which proposes that selection acts on the holobiont (host and associated microbes), and not simply on animals in isolation (Bordenstein and Theis, 2015).

Here, we utilize a replicated, multidirectional selection experiment on a non-laboratory, omnivorous rodent, the bank vole (*Myodes glareolus*), with lines selected in three directions: increased maximum rate of exercise-induced (swim) aerobic metabolism (Aerobic-selected), ability of young voles (32–36 days) to grow on a low-quality herbivorous diet (Herbivorous-selected), and intensity of predatory behavior toward crickets (Predatory-selected; Sadowska et al., 2008). Four lines in each of the three selection directions and four unselected (random bred) control lines (Control) were maintained.

We investigated whether the experimental evolution of phenotypic traits was paired with changes in gut microbial community structure. We collected contents from the cecal and foregut (from Herbivorous-selected and Control lines only) chambers and inventoried microbial communities by sequencing the 16S rRNA gene. Importantly, the individuals used in the analyses were never subjected to any phenotypic tests (such as the swim test, feeding on low-quality diets, or exposure to crickets). Thus, the low-quality diet or other factors resulting from the tests would not confound differences in microbial community structure.

It is well-known that herbivorous mammals harbor distinct and more diverse gut microbial communities, distinct from those of other mammals (Ley et al., 2008; Muegge et al., 2011) and the gut microbiome was important for the evolution of herbivory (Mackie, 2002; Stevens and Hume, 2004). Consequently, we predicted that Herbivorous-selected lines would harbor distinct cecal microbial communities from Control lines, and particularly more diverse microbial communities. We also predicted that the Herbivorous-selected lines might harbor communities with higher abundances of fermentative microbes (such as *Ruminococcus*). Further, the stomachs of voles have a foregut chamber anterior to the gastric chamber (Stevens and Hume, 2004). This foregut chamber houses a dense and active microbial community in other herbivorous rodents (Kohl et al., 2014a), and is thought to be important for the evolution of herbivory in rodents (Carleton, 1973). Therefore, we also predicted that the foregut communities of Herbivorous-selected voles would be distinct and more diverse when compared to Control lines.

Voles from the Aerobic-selected lines not only achieve a higher exercise-induced metabolic rate, but also have an increased basal metabolic rate (Sadowska et al., 2015) and daily food consumption (Dheyongera et al., 2016), which can be associated with changes in gut microbiome (Spor et al., 2011). A growing body of evidence indicates that the microbiome can also affect various aspects of animal behavior (Heijtz et al., 2011; Ezenwa et al., 2012). Therefore, we also investigated the cecal microbiome of the Aerobic- and Predatory-selected lines. However, due to the lack of comparative studies into the microbiota of animals with different metabolic rates or behaviors, we did not have any specific *a priori* hypotheses or predictions. Distinct microbial communities between Control lines and the Aerobic- or Predatory-selected lines would be suggestive that the microbiota is influenced by selection on the host-level phenotypes.

## Materials and methods

### Animals and the selection experiment

This work was performed on bank voles (*Myodes* = *Clethrionomys glareolus* Schreber 1780) from generation 15 of an ongoing artificial selection experiment maintained at the Jagiellonian University (Poland). While it would be interesting to compare the gut communities of generations prior to this, samples for microbiome analyses were not preserved until generation 15. The rationale, history and protocols of the experiment have been presented in our earlier work (Sadowska et al., 2008, 2015; Chrząścik et al., 2014; Konczal et al., 2015). Briefly, the colony was started from wild-caught individuals in 2000–2001, and for a few generations the voles were randomly mated and were used as a basis for quantitative genetic studies of metabolic traits (Sadowska et al., 2005, 2009). Then, the selection experiment was designed to mimic the adaptive radiation of mammals (Sadowska et al., 2008). The selection was applied based on the following criteria: High aerobic metabolism (Aerobic-selected)—the maximum 1-min rate of oxygen consumption (VO_2_ swim), achieved during 17-min of swimming at 38°C; Herbivorous capability (Herbivorous-selected)—body mass change in a 4 day trial, during which voles were fed a low-quality, herbivorous diet (made of dried grass powder, same as used as a component of standard diet, and flour); Predatory behavior (Predatory-selected)—ranked time to catch a live cricket in a 10-min trial (ranks 1–5: cricket caught in 0.5, 1, 3, 6, or 10 min, respectively; rank 6: cricket not caught). The measurements of swim-induced aerobic metabolism and the predatory behavior tests were performed on adults (about 75–95 days old), and the tests with low-quality diet on young, growing animals (32–36 days). All the trait values used as selection criteria were mass-adjusted (residuals from ANCOVA including also other covariates and cofactors). Four replicate lines for each selection direction and unselected control lines (C) were maintained (to allow valid tests of the effects of selection), with 15–20 reproducing families in each of the 16 lines (which avoided excess inbreeding).

The animals were maintained in standard plastic mouse cages with sawdust bedding, at a constant temperature (20 ± 1°C) and photoperiod (16:8 h light:dark; light phase starting at 2:00 am). Water and food (a standard rodent chow: 24% protein, 3% fat, 4% fiber; Labofeed H, Kcynia, Poland) was provided *ad libitum*. Animals were never co-housed across selection lines, but no sanitary barrier was maintained between the cages. The cages were of the open type (model 1264C or 1290D; Tecniplast, Bugugiatte, Italy), and voles from different lines were placed in the same rooms and often on the same racks, so dust containing feces and also fecal pellets were transferred (although not intentionally) between cages. No sanitary barrier was maintained during routine work, such as cage changes or weighing the animals (we did not change gloves or weighing dishes between handling subsequent animals). Thus, all the voles were practically exposed to microbes present in feces produced by all other voles.

Gut contents were sampled from 95 individuals (five or six individuals from each of the four replicate lines of four selection directions) of both sexes at the age of 148–182 days (mean: 166 days). Details regarding sample sizes, sex, and mean age for each line can be found in Supplementary Table 1. The individuals chosen for this project represented a random sample from respective lines and were not used in any physiological trials. Particularly, they were not used in the test with low-quality diet. All individuals came from different mothers and different cages. The animals were euthanized with CO_2_ in a chamber with gradually increasing CO_2_ concentration, and immediately dissected. Contents of caecum and the foregut (proximal chamber of stomach) were removed, preserved in RNAlater, and stored at −22°C for 4–5 months.

All the procedures were approved by the decision of the Local Ethical Committee for Experiments on Animals in Kraków, Poland (decisions No. 99/2006, 21/2010, and 22/2010).

### DNA sequencing

All samples were transported on dry ice to the University of Utah and stored at −80°C for 2 months. Whole DNA was extracted using a QIAamp DNA Stool Mini Kit (Qiagen, Germantown, MD). Extracted DNA was stored at −80°C for no more than 1 month, and was then shipped on dry ice to Argonne National Laboratories for sequencing. Bacterial inventories were conducted by amplifying the V4 region of the 16S rRNA gene using primers 515F and 806R and paired-end sequencing on an Illumina MiSeq platform (Caporaso et al., 2012). All sequences were deposited in the Sequence Read Archive (SRA) under accession PRJNA283286.

Sequences were analyzed using the QIIME software package (Caporaso et al., 2010). Sequences underwent standard quality control and were split in to libraries using default parameters in QIIME. The sequences were grouped into *de novo* operational taxonomic units (OTUs) using UCLUST (Edgar, 2010) with a minimum sequence identity of 97%. The most abundant sequences within each OTU were designated as a “representative sequence” and then aligned against Greengenes 13_5 (DeSantis et al., 2006) using PyNAST (Caporaso et al., 2009) with default parameters set by QIIME. Chimeric sequences were detected and removed using ChimeraSlayer (Haas et al., 2011). A PH Lane mask supplied by QIIME was used to remove hyper variable regions from aligned sequences. FastTree (Price et al., 2009) was used to create a phylogenetic tree of representative sequences. OTUs were classified using the Ribosomal Database Project classifier with the standard minimum support threshold of 80% (Wang et al., 2007). Singleton OTUs and sequences identified as chloroplasts or mitochondria were removed from analysis. Relative abundances of microbial phyla and genera were normalized using variance stabilizing transformation of arcsin (abundance^0.5^; Shchipkova et al., 2010; Kumar et al., 2012). We did not use rarefied data when comparing abundances of microbial taxa (McMurdie and Holmes, 2014).

### Data analysis

We compared several aspects of microbial diversity of cecal communities across all selection directions. First, we calculated the mean Shannon Index from 20 iterations of sub-sampling of 22,800 sequences from each sample. The Shannon Index was compared using pair-wise nested ANOVA comparing experimental selection lines with control lines. We used selection direction and selection line nested within selection direction as variables. We also compared community membership (the presence and absence of bacterial lineages) and community structure (taking relative abundances into account) of cecal communities across selection directions. We randomly selected 22,800 sequences per sample and then pooled them within a selection line in order to make each selection line an independent unit. We calculated unweighted (for community membership) and weighted (for community structure) Unifrac distances of the resulting pooled sequences (Ley et al., 2005). Principal coordinates analysis (PCoA) was used to visualize the similarities of these microbial communities. Community membership and structure were statistically compared using analysis of similarity (ANOSIM; Clarke, 1993). For nested ANOVAs and ANOSIM tests, we handled the pairwise comparisons differently. Given the strong *a priori* hypotheses regarding differences between the Control and Herbivorous-selected lines, we treated this as a “planned contrast,” for which correction for multiple tests is not required (e.g., Sokal and Rohlf, 1995). When comparing the Control lines to the Aerobic- and Predatory-selected lines, we did not have strong *a priori* hypotheses, and our analysis was largely exploratory. Thus, here we conducted Bonferroni adjustments, using a α-value of 0.025 for these comparisons.

Relative abundances of bacterial phyla and genera were compared using nested ANOVA with selection direction and selection line nested within selection direction as variables. *P*-values were corrected using False Discovery Rate correction for multiple comparisons (Benjamini and Hochberg, 1995). Due to the large number of comparisons and our limited number of independent units (selection lines rather than individuals), we also present near-significant trends (0.05 < *P* < 0.1).

We further investigated changes in the microbial communities of lines selected for herbivory by comparing foregut communities between control and herbivory-selected lines, and also comparing foregut communities to cecal communities within these lines. The data analysis was largely similar to cecal samples as described above. However, due to slightly lower return of sequences per sample from some foregut samples, we were limited to randomly choosing 14,000 sequences per sample.

## Results

**Figure 1:**
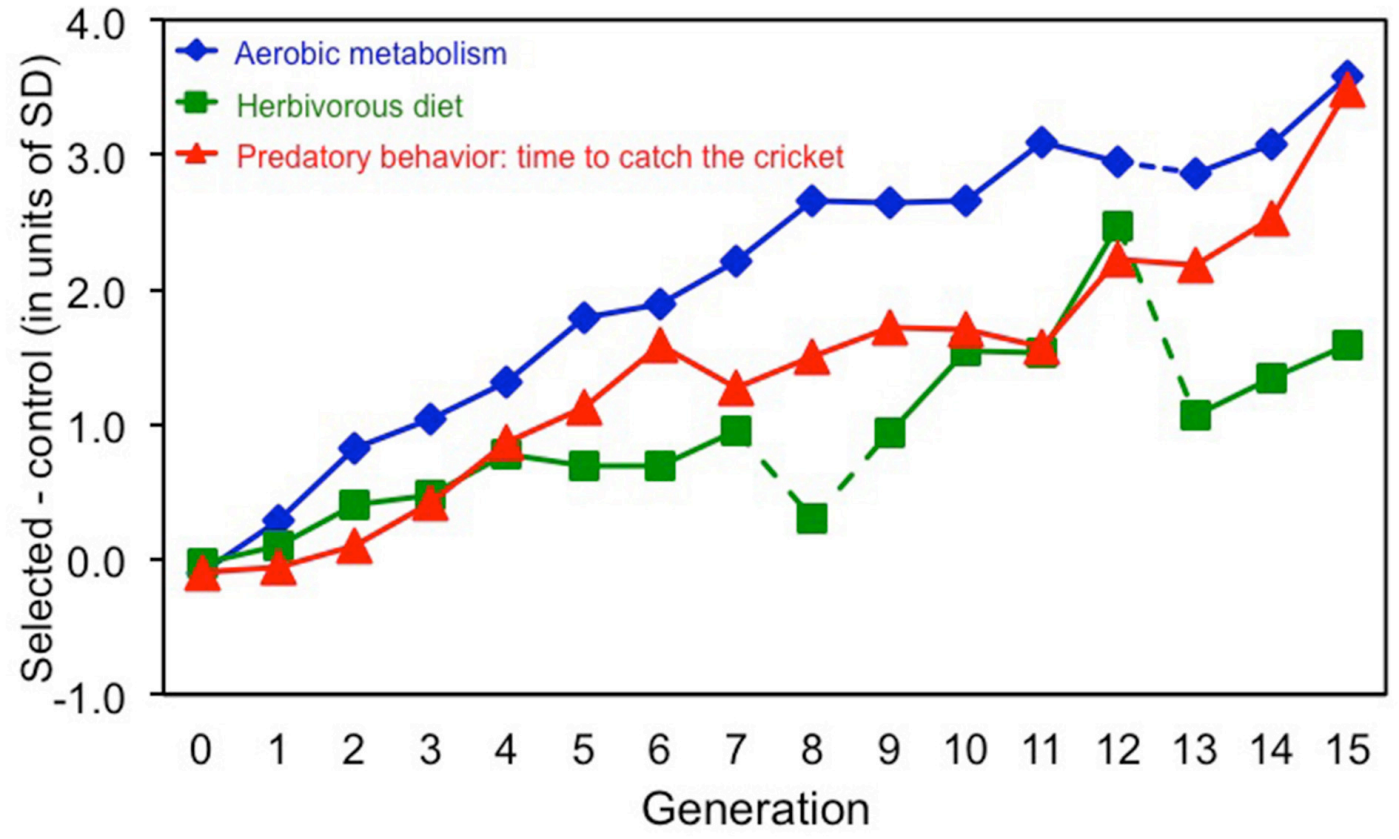
**Direct effects of 15 generations of selection on bank voles toward: high swim-induced aerobic metabolism, herbivorous capability measured as ability to maintain body mass on a low-quality diet, and predatory propensity measured as ranked time to attack a cricket**. Comparison of the cumulative effects of selection in the three directions is expressed as a difference between the means of four selected (in each direction) and four control lines, expressed in units of phenotypic standard deviation. In generation 8 and 13 the food composition used in selection trial in the “Herbivorous” lines was different than in other generations, which resulted in the irregular pattern (marked with broken lines). In generation 12 selection in “Aerobic” lines was relaxed (broken line).

In generation 15, voles from the Aerobic-selected lines achieved a 55% higher swim-induced maximum rate of oxygen consumption, those from the Herbivorous-selected lines lost 2.2 g less mass in the test with low-quality diet, and those from the Predatory-selected lines attacked cricket 7.7 times more frequently than voles from the unselected Control lines (Figure 1).

For cecal samples, we obtained an average of 62,988 ± 4301 high-quality sequences per sample. The number of sequences did not differ across selection directions [Nested ANOVA: Line nested within Selection Direction: *F*_(12, 79)_ = 0.76, *P* = 0.69; Selection Direction: *F*_(3, 12)_ = 0.05, *P* = 0.98]. Roughly 3500–3800 OTUs were identified per sample at a sampling depth of 22,800 sequences (Supplementary Figure 1).

**Figure 2:**
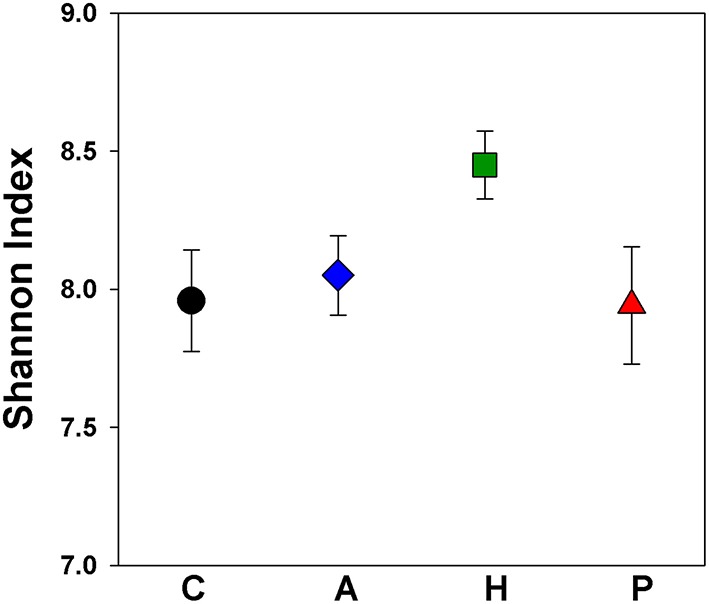
**Shannon index of microbial communities in the ceca of voles selected for different traits**. Points represent mean ± *SEM*. C, Control; A, Aerobic; H, Herbivorous; P, Predatory. Herbivorous-selected lines exhibited significantly higher biodiversity.

**Figure 3:**
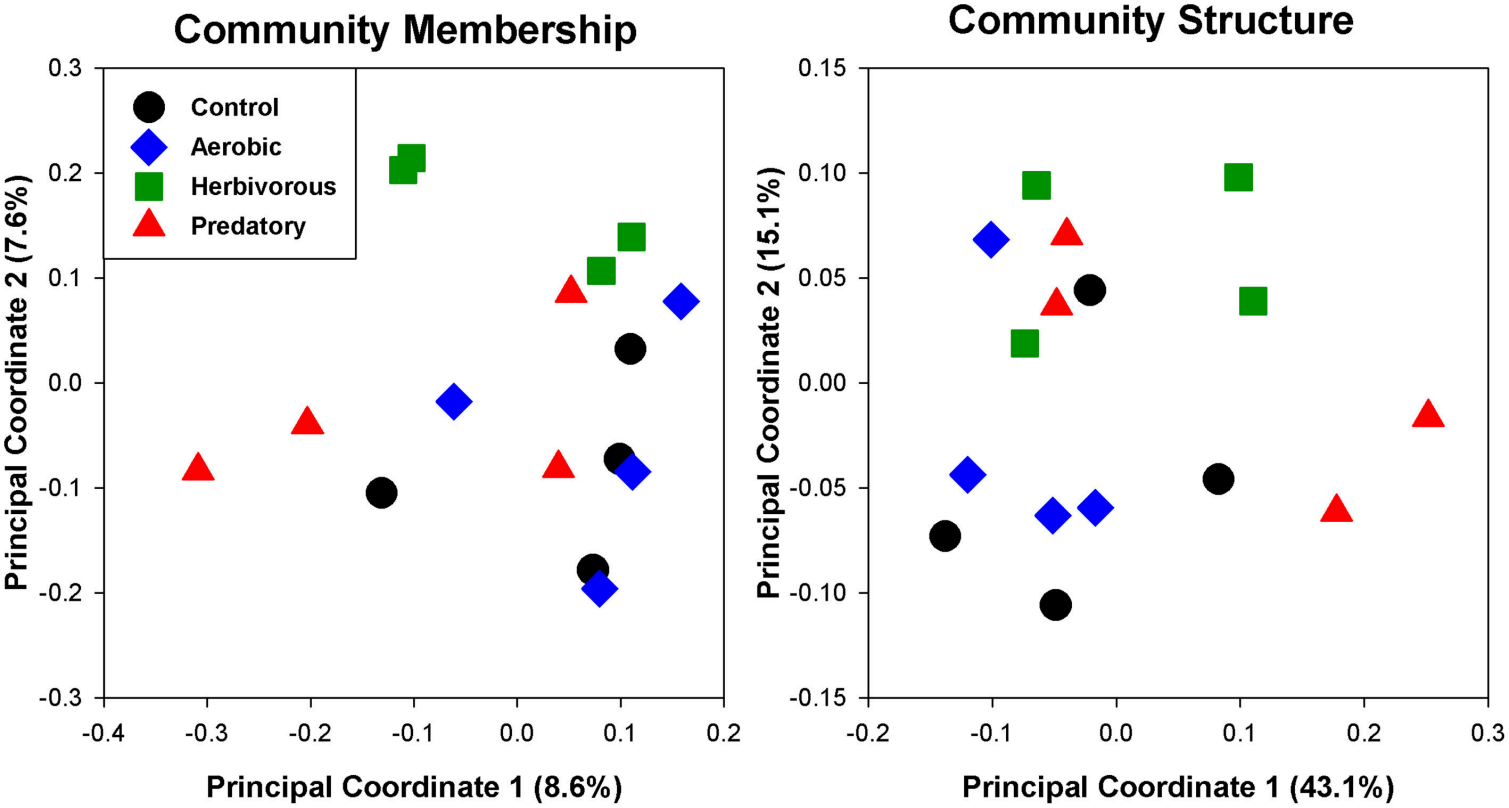
**Principal coordinate analyses of cecal communities of voles selected for various traits**. Community membership uses unweighted UniFrac distances, and thus investigates the presence or absence of bacterial lineages. Community structure uses weighted UniFrac distances, which take relative abundances of taxa into account.

The cecal chambers of Herbivorous-selected lines exhibited 6% higher microbial biodiversity, quantified with the Shannon index, than those of the Control lines [Figure 2; Nested ANOVA: Line nested within Selection Direction: *F*_(6, 40)_ = 0.66, *P* = 0.68; Selection Direction: *F*_(1, 7)_ = 4.76, *P* = 0.035]. There was no difference in the Shannon index between Control lines and Aerobic-selected or Predatory-selected lines (Effect of Selection Direction: *P* > 0.70 for both). The higher Shannon index of Herbivorous-selected cecal communities was driven by these voles harboring communities with higher community evenness [Nested ANOVA: Line nested within Selection Direction: *F*_(6, 40)_ = 0.73, *P* = 0.63; Selection Direction: *F*_(1, 7)_ = 4.98, *P* = 0.031], and to some extent higher observed microbial OTU (operational taxonomic unit) richness [Nested ANOVA: Line nested within Selection Direction: *F*_(6, 40)_ = 0.43, *P* = 0.85; Selection Direction: *F*_(1, 7)_ = 3.19, *P* = 0.082] compared to Control lines (Supplementary Figure 2). This pattern was also reflected in phylogenetic diversity, though the differences were not statistically significant (Supplementary Figure 1). The cecal communities of Herbivorous-selected lines also differed significantly from Controls in terms of community membership (Figure 3; ANOSIM: *R* = 0.45, *P* = 0.028), while Aerobic- and Predatory-selected lines did not differ from Controls (*P* > 0.3 for both). Selected lines did not exhibit significant differences from Control lines in community structure of cecal communities (Figure 3; ANOSIM: *P* > 0.15 for all).

When combining data across all groups, the cecal community was dominated by the phyla Firmicutes (56.7 ± 1.5% of community), Bacteroidetes (25.2 ± 1.7%), Spirochaetes (6.3 ± 0.8%), and Proteobacteria (5.8 ± 0.4%). The relative abundances of 16 additional phyla were all less than 1%. The most abundant identifiable genera were *Oscillospira* (5.8 ± 0.3% of community), *Treponema* (2.9 ± 0.3%), and *Ruminococcus* (2.1 ± 0.1%). We did not find any identifiable bacterial phyla or genera that exhibited significantly different or near-significant trends in relative abundances across selection directions for cecal communities.

For foregut samples, we obtained an average of 51,427 ± 5266 high-quality sequences per sample. There was no difference in the number of foregut sequences between Control and Herbivorous-selected lines and no significant difference between the number of sequences from foregut and cecal samples [Nested ANOVA: Line nested within Selection Direction: *F*_(6, 86)_ = 1.19, *P* = 0.32; Selection Direction: *F*_(1, 6)_ = 0.003, *P* = 0.95; Gut Region: *F*_(1, 86)_ = 1.79, *P* = 0.19].

**Figure 4:**
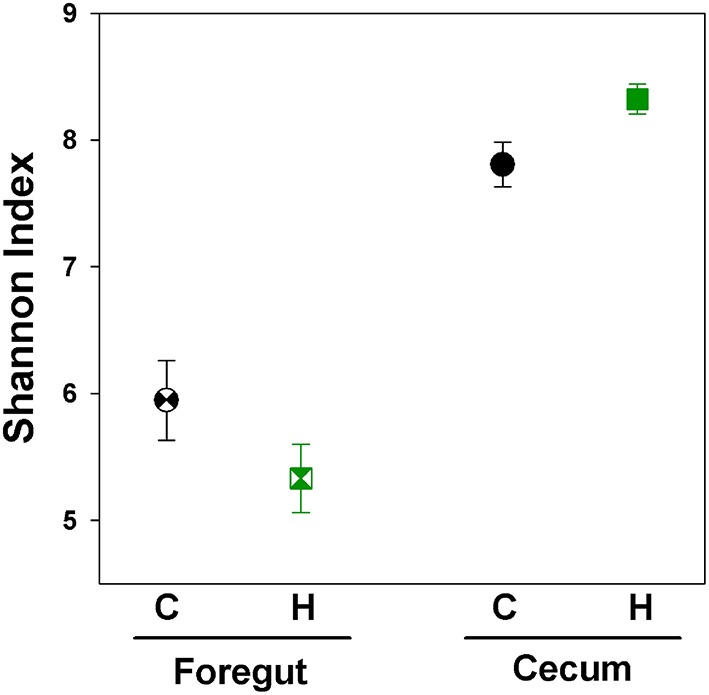
**Shannon index of microbial communities of voles selected for different traits**. Points represent mean ± *SEM*. C, Control; H, Herbivorous. The foregut chamber harbored lower microbial diversity than the cecal chamber. No difference in diversity was observed between the foregut communities of Control and Herbivorous-selected voles. Cecal communities of Herbivorous-selected lines were significantly more diverse than Control lines. The points for cecal samples represent the same samples as presented in Figure 2, except here we used a lower number of sequences per sample for analysis, due to a slightly lower return of sequences from some foregut samples.

Foregut samples harbored a lower diversity when compared with cecal samples [Figure 4; Nested ANOVA: Gut Region: *F*_(1, 86)_ = 103.27, *P* < 0.0001]. This analysis utilized the same cecal data as presented above, only utilizing a lower number of sequences for analysis, due to a slightly lower return of sequences from some foregut samples. When all Control and Herbivorous-selected samples were investigated together, there were no differences in diversity between selection directions or replicate lines [Line nested within Selection Direction: *F*_(6, 86)_ = 0.59, *P* = 0.73; Selection Direction: *F*_(1, 6)_ = 0.03, *P* = 0.86]. When only foregut samples were analyzed, there was no difference in the Shannon Index between Control and Herbivorous-selected lines [Nested ANOVA: Line nested within Selection Direction: *F*_(6, 41)_ = 0.66, *P* = 0.68; Selection Direction: *F*_(1, 7)_ = 2.06, *P* = 0.16].

**Figure 5:**
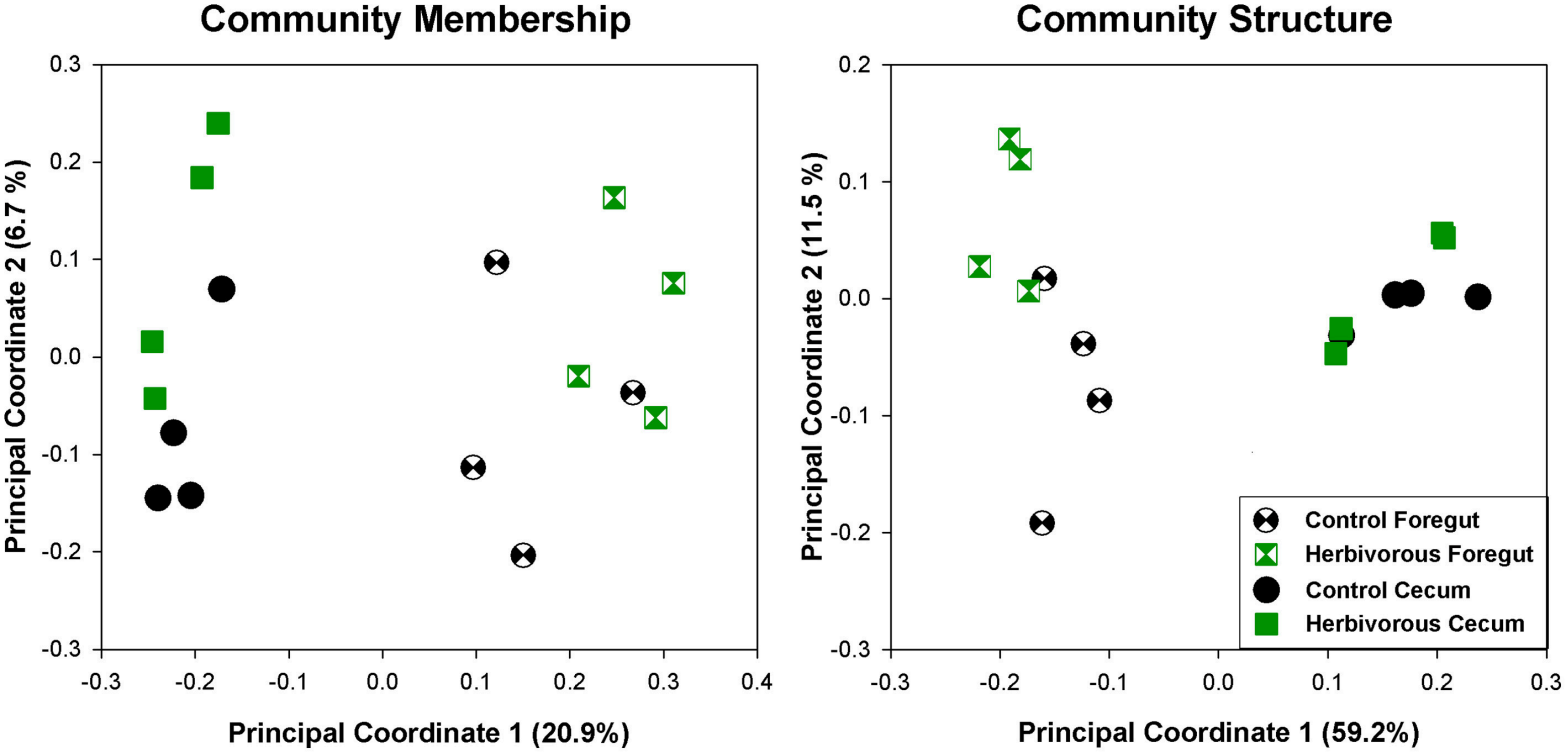
**Principal coordinate analyses of foregut and cecal communities of control and herbivorous-selected voles**. Community membership uses unweighted UniFrac distances, and thus investigates the presence or absence of bacterial lineages. Community structure uses weighted UniFrac distances, which takes relative abundances of taxa into account. The points for cecal samples represent the same samples as presented in Figure 3, except here we used a lower number of sequences per sample for analysis, due to a slightly lower return of sequences from some foregut samples.

Selection for the ability to cope with a high-fiber diet significantly altered the foregut microbial community in comparison to Control lines. Foregut communities of Herbivorous-selected lines exhibited significantly different microbial community membership (Figure 5; ANOSIM: *R* = 0.38, *P* = 0.05) and community structure (Figure 5; ANOSIM: *R* = 0.47, *P* = 0.04). However, cecal communities did not exhibit distinct microbial community membership (ANOSIM: *R* = 0.28, *P* = 0.11) or structure (*R* = 0.15, *P* = 0.26) in this analysis.

**Figure 6:**
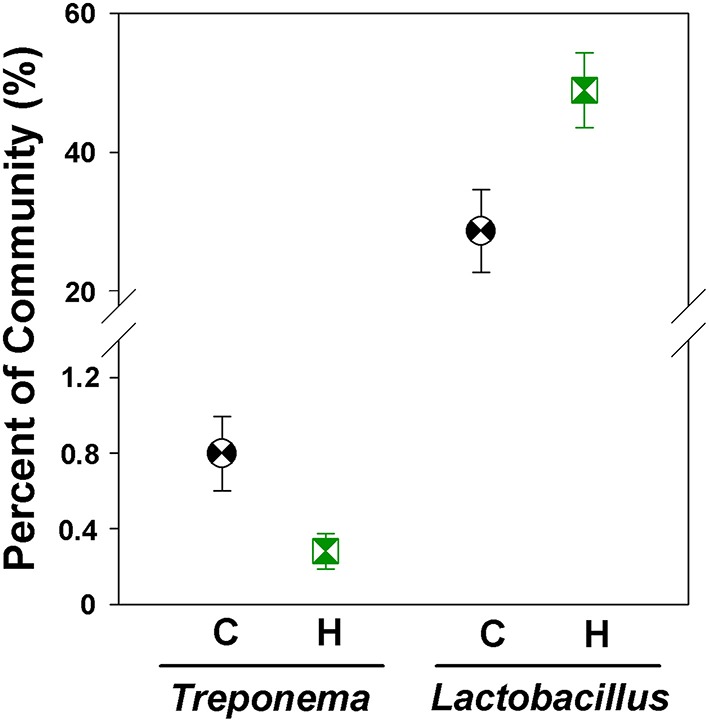
**Relative abundances of *Treponema* and *Lactobacillus* in the foregut chambers of control and herbivorous-selected voles**. Points represent mean ± *SEM*.

The foregut community was also dominated by Firmicutes (53.7 ± 4.0% of community), Bacteroidetes (30.9 ± 4.1%), and Proteobacteria (10.6 ± 1.7%). We did not detect any foregut microbial phyla that differed significantly in relative abundances between Control and Herbivorous-selected lines; however differences were detectable at lower taxonomic units. The microbial genus *Treponema* was significantly less abundant in the foregut of Herbivorous-selected lines compared to Controls [Figure 6; Nested ANOVA of transformed abundance data: Line nested within Selection Direction: *F*_(6, 45)_ = 0.68, *P* = 0.66; Selection Direction: *F*_(1, 6)_ = 8.09, FDR-correct *P*-value = 0.049]. There was also a near-significant trend for Herbivorous-selected lines to harbor a 1.7 × higher abundance of *Lactobacillus* compared to Control lines [Figure 6; Nested ANOVA of transformed abundance data: Line nested within Selection Direction: *F*_(6, 45)_ = 0.44, *P* = 0.85; Selection Direction: *F*_(1, 6)_ = 7.05, FDR-correct *P*-value = 0.065].

## Discussion

Here, we tested whether whole-organism selection for physiological and behavioral traits might be paired with changes in gut microbial community structure. We found that voles selected for herbivorous capability harbored distinct microbial communities (although, the differences were only marginally significant), while voles selected for high aerobic capacity or predatory behavior exhibited no differences from control lines.

We did not observe any differences in cecal microbial community structure between Control lines and Aerobic-selected or Predatory-selected lines. Germ-free animals have lower basal metabolic rate, heart size, cardiac output, hematocrit, and overall blood volume compared to conventional animals (Gordon et al., 1963; Bäckhed et al., 2004; Crawford et al., 2009). Therefore, one might postulate that evolution of microbiome communities could accompany the evolution of metabolic performance traits. Similarly, the microbiome has been implicated as a mechanism in the evolution of various animal behaviors (Ezenwa et al., 2012). Though, Ezenwa et al. (2012) acknowledge that not all animal behaviors are likely to influence or be influenced by the microbiome. Determining which behaviors are associated with the microbiome will help us in understanding the potential and limitations for microbes to serve as drivers of behavioral evolution. Our study suggests that both higher exercise-induced metabolic rates and predatory behavior could evolve independently of alterations in gut microbial community structure. Though, it should be noted that we only inventoried taxonomic diversity, and there may be underlying changes in the abundance of genes with associated functions that would be uncovered by metagenomic sequencing.

Conversely, the Herbivorous-selected lines harbored more diverse cecal microbial communities when compared to Control lines. These findings are consistent with comparative studies, which demonstrate that a more diverse microbial community is a trait of herbivorous mammals (Ley et al., 2008). This higher diversity has largely been attributed to the higher fiber content of diets of herbivorous mammals (Ley et al., 2008) and the fact that diet has a large influence on microbial community structure (Carmody et al., 2014). Our results suggest that a more diverse microbial community may be an evolved trait of mammalian herbivores, given that the animals in our study were all feeding on the same nutrient-rich rat chow and had never been exposed to an herbivorous diet (below we discuss an alternative explanation of the results). Prior studies have found positive correlation between the taxonomic diversity of a microbial community and its functional diversity (HMP Consortium, 2012); if this holds true in our system, the Herbivorous-selected vole microbiota may have more diverse functions compared to control lines. Additionally, when comparing microbiomes across mammalian species, taxonomically distinct microbial communities encode functionally distinct repertoires of enzymes active against carbohydrates (Muegge et al., 2011). Thus, the microbiota of Herbivorous-selected voles may be functionally diverse and distinct compared to Control voles, which likely aid them in coping with high-fiber diets. Alternatively, the microbiota can influence systemic nutrient routing and storage by the host (Bäckhed et al., 2004; Crawford et al., 2009). Since the selection metric for our study is maintenance of body mass on a high-fiber diet, the distinct microbial communities hosted by Herbivorous-selected lines may be solely associated with increasing maintenance of body mass. Further physiological studies could elucidate the functions provided by these communities.

The community structure in the foregut chamber also differed between Control and Herbivorous-selected lines of voles. This chamber has long been suggested to be important for the evolution of herbivory in rodents (Carleton, 1973; Toepfer, 1891). Other herbivorous rodents (*Neotoma* spp.) house dense microbial communities that produce high concentrations of volatile fatty acids within the foregut chamber (Kohl et al., 2014a). Our results showed that selection for herbivory altered the microbial community structure of this chamber, including changes in the relative abundances of several taxa. The genus *Treponema* exhibited lower abundances in the foreguts of Herbivorous-selected voles compared to Controls. While members of *Treponema* do not degrade cellulose, they can degrade other plant polysaccharides (Paster and Canale-Parola, 1985; Piknova et al., 2008) and their presence enhances fiber fermentation by other cellulolytic bacteria (Kudo et al., 1987). Thus, it is somewhat puzzling why abundances of this fermentation-enhancing genus would be lower in the Herbivorous-selected lines compared to Controls. However, *Treponema* may perform different functions in the unique chamber of the rodent foregut. It would be very interesting to compare how the microbial communities of Control and Herbivorous-selected lines change when placed on a high-fiber diet.

We also observed a near-significant increase in the abundance of *Lactobacillus* in the foregut chambers of herbivorous-selected voles. *Lactobacillus* is the dominant genus in the foregut chambers of other herbivorous rodents (Kohl and Dearing, 2012; Kohl et al., 2014a; Shinohara et al., 2016). It is unlikely that members of *Lactobacillus* are conducting extensive fiber fermentation in the foregut chamber, given they are not largely cellulolytic and residence time of food material in this chamber is very short (Kohl et al., 2014a). This microbial population could be important for recycling urea nitrogen, given that the rodent foregut contains high concentrations of ammonia nitrogen (Kohl et al., 2014a). Nitrogen recycling may improve animals' nitrogen economy, thus allowing them to maintain body mass on a low-protein food (Stevens and Hume, 2004).

Several mechanisms may underlie how Herbivorous-selected voles come to harbor distinct and diverse communities. First, the microbiome is easily altered by diet (Carmody et al., 2014) and is transmitted from mother to offspring (Funkhouser and Bordenstein, 2013; Kohl et al., 2014b). It could be argued that mothers simply acquire from the experimental diet some microbes not present in the standard food, and then transmit it to the offspring. However, a controlled feeding trial in wood rats (*Neotoma* spp.) found that the microbial species present in laboratory animal chows accounted for only 0.5% of the fecal microbiota (Kohl and Dearing, 2014). Additionally, exposure to the high-fiber diet might alter the microbiota of mothers, which in turn affects which microbes are transmitted to offspring (Sonnenburg et al., 2016). Though, an elegant study in humanized-mice (germ-free mice colonized with human microbiota) demonstrated that founder effects can be important for initial community structure, but that current diet supersedes these effects over time (Turnbaugh et al., 2009). Mothers in our selection experiment are fed the high-fiber diet for 4 days from the age of 32–36 days, and are then fed basic rodent chow for roughly 2.5 months before reproducing. Thus, it is unlikely that the transient effect of the high-fiber diet impacts which microbes are transmitted from mother to offspring, and that the altered composition is maintained in the offspring for the next 5 month, especially given that the gut microbiota rapidly responds to changes in diet (David et al., 2014a,b). Moreover, because of the lack of any sanitary barrier between cages, all voles in the colony were permanently exposed to the same load of microbes dispersed in feces or dust containing dried fecal particles. Rather, it is likely that there is selection within the microbial communities for members that aid in digestion or maintenance of body mass of the hosts. However, to completely render the possibility of transient effects not related to the selection, further experiments should be performed to check whether the microbiome differences are maintained also when mothers from Control lines are fed the experimental diet or are co-housed with those from the H-selected lines.

Distinct microbial communities could also be brought about by host evolution. For example, changes in sequences or expression profiles of epithelial-produced glycans (Hooper and Gordon, 2001), antimicrobial peptides (Franzenburg et al., 2013), or other genes (Spor et al., 2011) may drive changes in microbial community structure. Future studies could investigate the mechanisms that facilitate these evolved changes in microbial community structure. Fortunately, continued selection experiments provide further generations of animals to test these questions. It would be especially interesting to correlate changes in microbial community structure across generations with the differential performance metrics between control and selected lines. For example, do the Herbivorous-selected individuals with higher microbial diversity exhibit higher performance on the low-quality diet?

Understanding the evolution of mammalian herbivory is important, given that herbivory is the most common feeding strategy among mammals (Price et al., 2012), and herbivores can play large roles in determining the structure of entire ecosystems (Martin and Maron, 2012). The evolution of herbivory is thought to be largely facilitated by mutualistic relationships with gut microbes that aid in the digestion of fiber (Stevens and Hume, 2004) and the degradation of plant toxins (Kohl et al., 2014c). Here we have demonstrated that omnivorous rodents selected for the ability to maintain body mass on a fiber-rich diet maintain distinct and more diverse gut microbial communities independent of current diet. This work supports the “hologenome concept” by demonstrating that selection on a host performance metric can result in changes in the associated microbiota (Bordenstein and Theis, 2015). Importantly, the “hologenome concept” assumes that these changes can be driven by the genetics of the host, the microbiota, or both (Theis et al., 2016). Therefore, further work is needed to check whether the differences in microbial composition are robust to environmental factors, to uncover the functions provided by these microbial communities, and understand the mechanistic basis underlying their community assembly. We argue that our selection experiment is a promising tool to further investigate the role of gut microbes in facilitating the evolution of herbivory in mammals and their broader functions in ecology and evolution.

## Author contributions

KK performed microbial inventories and data analysis, and wrote the paper. ES and AR conducted the selection experiment, collected samples, and assisted with revisions of the paper. MD and PK helped with interpretation of data, oversaw the project, and revised the paper. All authors approve the final version for publication.

## Funding

This research was supported by the National Science Foundation (Doctoral Dissertation Improvement Grant, DEB 1210094, to MD and KK; DEB 1342615 to MD; and DBI 1400456 to KK) and the Polish National Science Centre (NN 303 816740 to PK) and the Jagiellonian University (DDS/WBINOZ/INOS/757 to PK).

### Conflict of interest statement

The authors declare that the research was conducted in the absence of any commercial or financial relationships that could be construed as a potential conflict of interest.
