# Supplementary material for: Experimental Evolution on a Wild Mammal Species Results in Modifications of Gut Microbial Communities
Source: Front Microbiol. 2016 May 4;7:634. doi: 10.3389/fmicb.2016.00634 (PMC4854874; doi:10.3389/fmicb.2016.00634)
Supplement: Supplementary file 1 [file Table1.PDF]

**Suppl. Table 1**. Details regarding sample size, sex, and age of voles in each group.

| Selection Direction | **Control** | | | | **Aerobic** | | | | **Herbivorous** | | | | **Predatory** | | | |
|---|---|---|---|---|---|---|---|---|---|---|---|---|---|---|---|---|
| Line | **C1** | **C2** | **C3** | **C4** | **A1** | **A2** | **A3** | **A4** | **H1** | **H2** | **H3** | **H4** | **P1** | **P2** | **P3** | **P4** |
| N (males/females) | 6 (5/1) | 6 (3/3) | 6 (4/2) | 6 (2/4) | 6 (3/3) | 6 (3/3) | 6 (4/2) | 6 (4/2) | 6 (2/4) | 6 (2/4) | 6 (4/2) | 6 (3/3) | 5 (3/2) | 6 (3/3) | 6 (2/4) | 6 (2/4) |
| Mean age in days (± s.e.m.) | 164.2 ± 5.0 | 169.8 ± 3.8 | 167.7 ± 3.8 | 165.8 ± 4.8 | 166.8 ± 4.4 | 165.8 ± 4.6 | 163.2 ± 4.3 | 165.5 ± 3.7 | 164.7 ± 3.9 | 165.3 ± 4.5 | 164.3 ± 5.9 | 166.3 ± 4.9 | 167.2 ± 4.3 | 170.0 ± 4.6 | 166.0 ± 3.3 | 168.2 ± 4.6 |
