# Supplementary figures and images for: Experimental Evolution on a Wild Mammal Species Results in Modifications of Gut Microbial Communities
Source: Front Microbiol. 2016 May 4;7:634. doi: 10.3389/fmicb.2016.00634 (PMC4854874; doi:10.3389/fmicb.2016.00634)

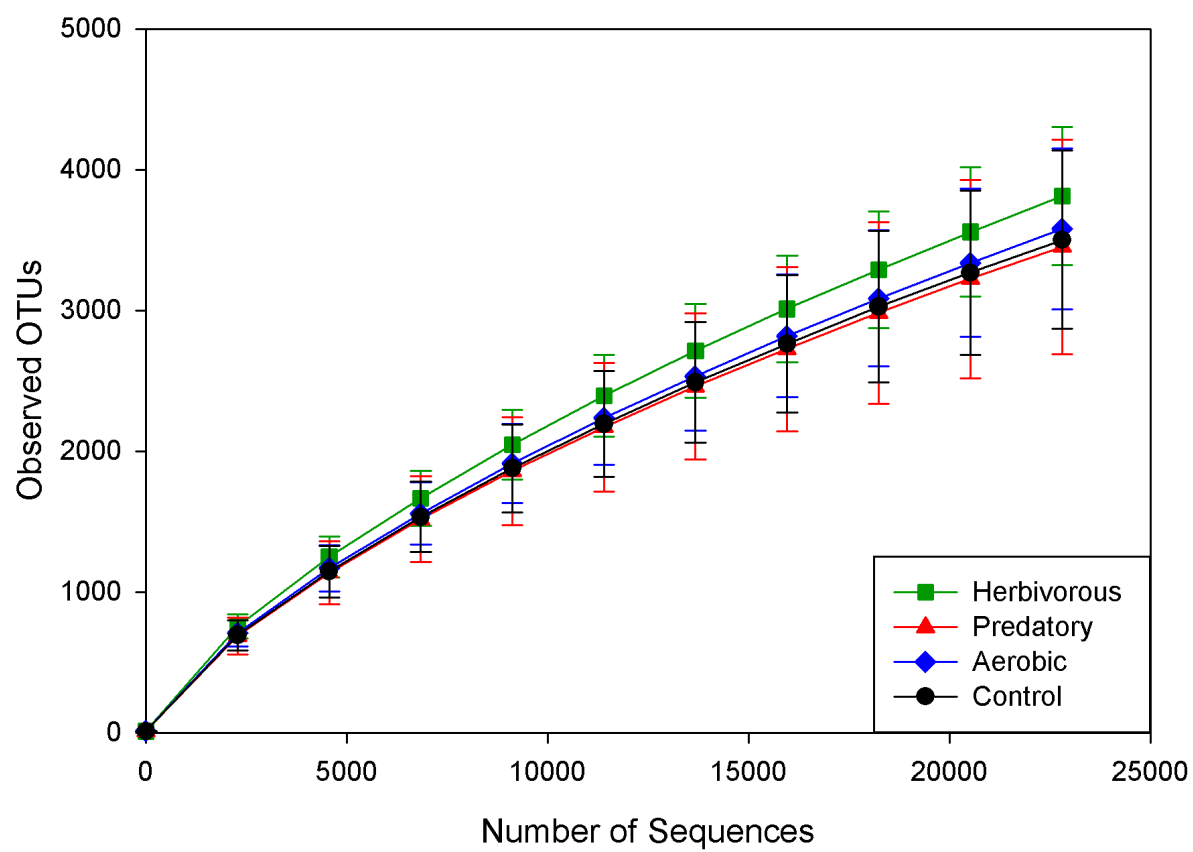

**Suppl. Fig. 1.** Rarefaction curves for number of observed OTUs per sample.

Supplement: Supplementary file 2 [file Image1.PDF]
