# Supplementary material for: Experimental Evolution on a Wild Mammal Species Results in Modifications of Gut Microbial Communities
Source: Front Microbiol. 2016 May 4;7:634. doi: 10.3389/fmicb.2016.00634 (PMC4854874; doi:10.3389/fmicb.2016.00634)
Supplement: Supplementary file 3 [file Image2.PDF]

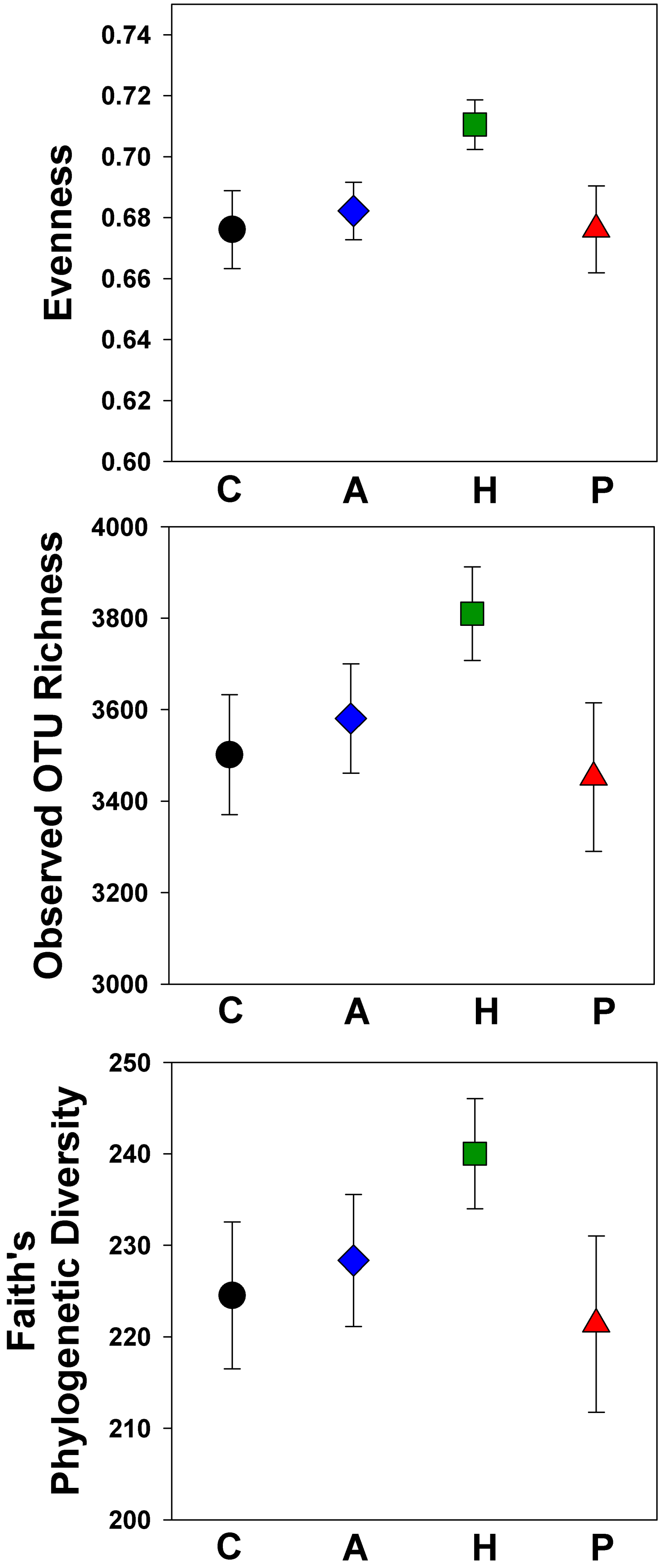

Suppl. Fig. 2: Biodiversity measurements of microbial communities in the ceca of voles selected for different traits. Points represent mean ± SEM. C: Control; A: Aerobic; H: Herbivorous; P: Predatory.
